# Low cholesterol levels are associated with increasing risk of plasma cell neoplasm: A UK biobank cohort study

**DOI:** 10.1002/cam4.6649

**Published:** 2023-11-01

**Authors:** Linfeng Li, Zhengyu Yu, Jianjun Ren, Ting Niu

**Affiliations:** ^1^ Department of Hematology, Institute of Hematology, West China Hospital Sichuan University Chengdu China; ^2^ Department of Otolaryngology‐Head and Neck Surgery, West China Hospital, West China Medical School Sichuan University Chengdu China

**Keywords:** apolipoprotein, cholesterol, plasma cell neoplasm, UK Biobank

## Abstract

**Background:**

Plasma cell neoplasms are a group of hematologic neoplasms that often develop in the elderly population. The relationship between cholesterol levels and hematologic malignancy has been identified in population studies. However, it is still unclear if there is a relationship between cholesterol levels and plasma cell neoplasm in European ancestry.

**Methods:**

Prospective cohorts included 502,507 individuals from the UK Biobank who were followed up to 2019 and assessed total cholesterol(TC) levels, low‐density lipoprotein (LDL) levels, high‐density lipoprotein (HDL) levels, apolipoprotein A (ApoA) and apolipoprotein B (ApoB) as risk factors for plasma cell neoplasms with Cox proportional hazard regression and restricted cubic spline model. We also used two‐sample Mendelian randomization to determine if the cholesterol level has a causal effect on developing plasma cell neoplasms.

**Results:**

We observed 1819 plasma cell neoplasm cases during 14.2 years of follow‐up in the UK Biobank. We found higher blood serum cholesterol levels at baseline were associated with a lower risk of plasma cell neoplasm in our study. All lipid profiles we analyzed in this study were inversely associated with plasma cell neoplasm risk (all *p*_trend_ <0.005) but triglycerides did not have such association. However, there was no suggestive association of genetically predicted serum LDL, HDL, and total cholesterol levels with multiple myeloma.

**Conclusion:**

Low serum total cholesterol, LDL, HDL, ApoA, and ApoB levels were all associated with increasing the risk of plasma cell neoplasm.

## Graphical abstract

We found that cholesterol profiles such as total cholesterol, HDL, LDL, apolipoprotein A, and apolipoprotein B were inversely correlated with the risk of plasma neoplasms.

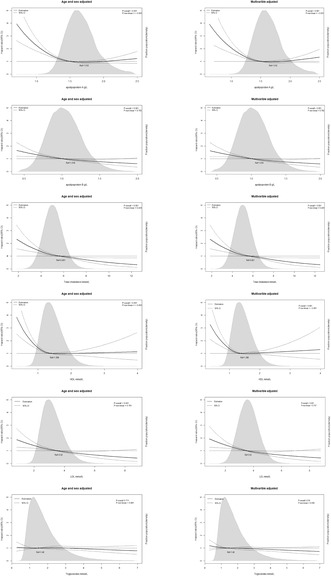

## 1 INTRODUCTION

Plasma cell neoplasms are a group of diseases originating from terminally differentiated B‐cells and characterized by the secretion of monoclonal immunoglobulin in most cases. These disorders have a common pathway of development from a benign condition called monoclonal gammopathy of unknown significance (MGUS) and are usually asymptomatic with minimal evidence of disease. Multiple myeloma (MM) and solitary plasmacytomas are malignant plasma cell neoplasm disorders resulting from clonal plasma cell expansion. MM is the second most common hematologic malignancy, accounting for 2% of all cancer deaths and MGUS patients can progress to MM and related dyscrasia with a sustained rate of 1% per year.^1^, ^2^, ^3^, ^4^

Dysregulation of lipid metabolism often occurs in the elderly and has recently been implicated in the development and growth of cancer cells. Serum lipids and lipoproteins have been shown in experimental studies to potentially promote carcinogenesis through pathways involving insulin resistance, inflammation, antiapoptotic, and oxidative stress.^5^, ^6^, ^7^, ^8^ Epidemiological studies have revealed that LDL is closely associated with lung cancer,^9^ colorectal cancer,^10^ hematologic malignancies,^11^ and other types of cancer.^8^, ^12^, ^13^ Moreover, ApoB the component of LDL was considered as bad particle in cardiovascular disease and has been found to be associated with in short life span and several cancers risks.^14^ However, HDL level has been found to be inversely related to endometrial, colorectal, and lung cancer risks. In addition, some studies have found that people with hematologic malignancy such as MM are more likely to be accompanied by low levels of HDL, which may be an early sign of the disease.^15^, ^16^

Recently, a Korean population study found that individuals with lower baseline levels of HDL, as well as greater variability in HDL, were more likely to develop MM. These findings support the risk role of lipid metabolism dysregulation in MM.^17^ ApoA was an important component of HDL and now is considered to have multiple beneficial functions in cancer besides atherosclerosis, thrombosis, and diabetes.^18^ A large population study also found that the level of ApoA showed a similar result as HDL in cancer risks.^15^

Although some researches have indicated there might be a relationship between levels of lipid profiles and hematologic malignances, studies were mainly focused on the impact of HDL level. Our study was designed to investigate the relationship between various cholesterol metabolism parameters with overall plasma cell neoplasms in the prospective UK Biobank cohort study. We also investigated the potential causal relationship between plasma cell neoplasms and these metabolites levels using two‐sample MR analyses with summary genome‐wide association study (GWAS) data from UK Biobank and IEU Open GWAS project.

## 2 MATERIALS AND METHODS

### 2.1 Study population and study design

The UK Biobank, a large prospective cohort study, recruited a total of 502,507 people in 2006–2010. All these people were aged 40 to 69 years and registered with the UK National Health Service. The baseline data and characteristics of the population were collected by using touch‐screen questionnaires and verbal interviews with a trained nurse in an assessment Center. Participants’ blood was collected and serum was separated according to protocol in Companion document for serum biomarker data provided by UK Biobank, then aliquoted and stored in a central working archive at −80°C. The serum concentration of HDL, LDL, ApoA, ApoB, and triglyceride was measured in 99.7% of the population. This lipid metabolite was determined by Immuno‐turbidimetric assay with Beckman Coulter AU5800. Full details of the assay methods of serum biomarker data can be found in the UK Biobank biomarker project. (https://biobank.ndph.ox.ac.uk/showcase/refer.cgi?id=1227) HDL/LDL, HDL/ApoA, LDL/ApoB, and ApoB/ApoA ratios were also calculated and all components of the ratios were converted to the same unit before dividing.

Eligible participants in our study were participants aged 40 to 69 years at assessment who had no record of prior any cancer in national cancer registries except C44: nonmelanoma skin cancer) (*n* = 25,802). Participants with no serum lipid biomarker assessment (including HDL, LDL, ApoA, ApoB, and TC) or body mass index (BMI) were also excluded (*n* = 63,215). In order to reduce the potential influence of reverse causation on our study results, we also excluded participants within one year of follow‐up time (*n* = 4775). For sensitivity analysis, we further excluded participants within three years of follow‐up time (*n* = 4887) (Supplementary Figure S1).

The outcome was defined as the first diagnosis of any types of plasma cell neoplasms according to the International Classification of Disease, 10th Revision (ICD‐10) codes C90.0 (Multiple myeloma), C90.1 (Plasma cell leukemia), C90.2 (Plasmacytoma, extramedullary), C90.3 (Solitary plasmacytoma), C88.0 (Waldenstrom's macroglobulinemia), E85 (Amyloidosis) and C47.2 (monoclonal gammopathies).

### 2.2 Statistical analysis

In the analysis of baseline characteristics, we used proportions for categorical variables and median and interquartile range (IQR) for continuous variables. The Wilcoxon test and the Pearson test were then used to evaluate differences between cases and noncases for continuous variables. For categorical and continuous data, the Pearson and Wilcoxon tests were employed to evaluate differences between cases and noncases.

Our study conducted Cox proportional hazards models to investigate the relationship between plasma cell neoplasms diagnosis and lipid metabolism indicators. Hazard ratios (HRs) and 95% confidence intervals (CIs) were calculated in either univariate or multivariate Cox regression models. Lipid metabolism indicators are the primary exposure variables considered in our analyses. To describe potential nonlinear relationships between the variables and outcome, we use restricted cubic spline models placing four knots (5%, 35%, 65%, and 95%) according to Harrell's recommended percentiles by using the R package ‘rms’. We regarded the median values of lipid metabolites as a reference. Analyses were stratified by age at recruitment, Townsend deprivation index, metabolic equivalent task (MET) scores, ethnic group (Asian, black, white, mixed background, other and unknown), BMI, cigarette smoking (never, former, and unknown status), alcohol taking frequency (never, median (frequency less than twice a week), heavy (frequency over three times a week), unknown), and diabetes diagnosis (no, yes and unknown). Adjustment covariates were defined beforehand according to previous analyses of UK Biobank data.

The lipids parameters were also modeled as quantile grouping in the regression analyses. The P for trend was calculated by using the median of each exposure as a ranked variable in Cox models. Some of participants (<10%) had missing values for Townsend deprivation index and MET scores. The missing data were assumed to be random and were imputed using a multiple imputation method by chained equations.^19^ Five imputed data sets were created using 30 iterations. In order to get lowest bias and highest power in following survival analyses, we also included the Nelson‐Aalen estimator included in the imputation algorithm.^20^ Imputed data set was combined according to Rubin's rules.^21^ Cox proportional hazards modeling was done in R (Version 4.1.2) utilizing the ‘survival’ package.^22^

### 2.3 Two‐sample Mendelian randomization

Single‐nucleotide polymorphisms (SNPs) associated with total cholesterol, HDL‐C, LDL‐C, and ApoA were identified based on a publicly available GWAS from 3 different consortiums in the IEU Open GWAS project.^23^, ^24^, ^25^

Summary statistics were performed for SNP associations with multiple myeloma risk generated from the UK Biobank, which included 601 multiple myeloma cases and 372,016 controls of European ancestry. A two‐sample Mendelian randomization study was conducted to assess the potential association of circulating lipid metabolites with overall multiple myeloma risk, using UK Biobank and summary results from the IEU Open GWAS project as our source of genetic instruments for HDL, LDL, total cholesterol, and Apolipoprotein A. Then we used the ‘TwoSampleMR’ R package(R version 4.1.2) to perform MR analyses. All tests of significance were two‐sided, and *p*‐values <0.05 were considered statistically significant.

## 3 RESULTS

### 3.1 Population baseline

Among 408,722 individuals from the UK Biobank study, 1819 (0.45%) were diagnosed with plasma cell neoplasms during up to 14.2 years of follow‐up (median 12.2 years). (Table 1).

**TABLE 1:** Baseline characteristics of individuals in UK Biobank.

Characteristics	All subjects (*N* = 408,722)	Plasma cell neoplasms case (*N* = 1819)	Noncase (*N* = 406,903)	*p* ^a^
Age	58.00 [50.00, 63.00]	62.00 [56.00, 66.00]	58.00 [50.00, 63.00]	<0.001
Sex				<0.001
Male	189,547 (46.4)	1007 (55.4)	188,540 (46.3)	
Female	219,175 (53.6)	812 (44.6)	218,749 (53.7)	
Albumin	45.21 [43.51, 46.94]	44.68 [42.76, 46.46]	45.21 [43.51, 46.94]	<0.001
ApoA	1.51 [1.35, 1.70]	1.48 [1.30, 1.67]	1.51 [1.35, 1.70]	<0.001
ApoB	1.02 [0.86, 1.18]	0.98 [0.82, 1.15]	1.02 [0.86, 1.18]	<0.001
Total cholesterol	5.65 [4.91, 6.42]	5.42 [4.67, 6.27]	5.65 [4.91, 6.42]	<0.001
HDL	1.40 [1.17, 1.67]	1.34 [1.11, 1.64]	1.40 [1.17, 1.67]	<0.001
LDL	3.52 [2.95, 4.12]	3.36 [2.78, 4.01]	3.52 [2.95, 4.12]	<0.001
Triglycerides	1.48 [1.04, 2.14]	1.49 [1.05, 2.15]	1.48 [1.04, 2.14]	0.869
TDI	−2.15 [−3.65, 0.51]	−2.19 [−3.73, 0.24]	−2.15 [−3.65, 0.51]	0.155
MET	1779 [810, 3573]	1584 [716, 3573]	1779 [810, 3573]	0.026
BMI				0.002
<18.5	2078 (0.5)	9 (0.5)	2069 (0.5)	
18.5–24.9	132,703 (32.5)	519 (28.5)	132,184 (32.5)	
25–29.9	174,313 (42.6)	798 (43.9)	173,515 (42.6)	
≥30	99,628 (24.4)	493 (27.1)	99,135 (24.4)	
Alcohol taking frequency^b^				0.007
No	78,868 (19.3)	402 (22.1)	78,466 (19.3)	
Median	150,847 (36.9)	653 (35.9)	150,194 (36.9)	
Heavy	178,135 (43.6)	757 (41.6)	177,378 (43.6)	
Unknown	872 (0.2)	7 (0.4)	865 (0.2)	
Smoking status				0.008
Never	223,099 (54.6)	928 (51.0)	222,171 (54.6)	
Previous	140,330 (34.3)	691 (38.0)	139,639 (34.3)	
Current	43,274 (10.6)	189 (10.4)	43,085 (10.6)	
Unknown	2019 (0.5)	11 (0.6)	2008 (0.5)	
Qualification				0.054
Nonuniversity degree	271,301 (66.4)	1245 (68.4)	270,056 (66.4)	
University degree	132,622 (32.4)	547 (30.1)	132,075 (32.5)	
Unknown	4799 (1.2)	27 (1.5)	4772 (1.2)	
Diabetes				<0.001
No	386,020 (94.4)	1664 (91.5)	384,356 (94.5)	
Yes	20,950 (5.1)	144 (7.9)	20,806 (5.1)	
Unknown	1752 (0.4)	11 (0.6)	1741 (0.4)	
Ethnicity				<0.001
White	385,186 (94.2)	1689 (92.9)	383,497 (94.2)	
Black	7248 (1.8)	57 (3.1)	7191 (1.8)	
Asian	9156 (2.2)	41 (2.3)	9115 (2.2)	
Mix	5216 (1.3)	16 (0.9)	5200 (1.3)	
Unknown	1916 (0.5)	16 (0.9)	1900 (0.5)	
Cholesterol‐lowering medication				<0.001
Yes	70,750 (17.3)	452 (24.8)	70,298 (17.3)	
No	337,972 (82.7)	1367 (75.2)	336,605 (82.7)	

^a^ *p*‐values were determined from Wilcoxon rank‐sum test to compare differences for continuous variables and Pearson test for categorical variables between cases and noncases.

^b^ Median alcohol taking frequency is defined as frequency less than twice a week and heavy alcohol taking frequency is defined as frequency over three times a week.

### 3.2 Baseline cholesterol traits and risk of plasma cell neoplasms

**FIGURE 1:**
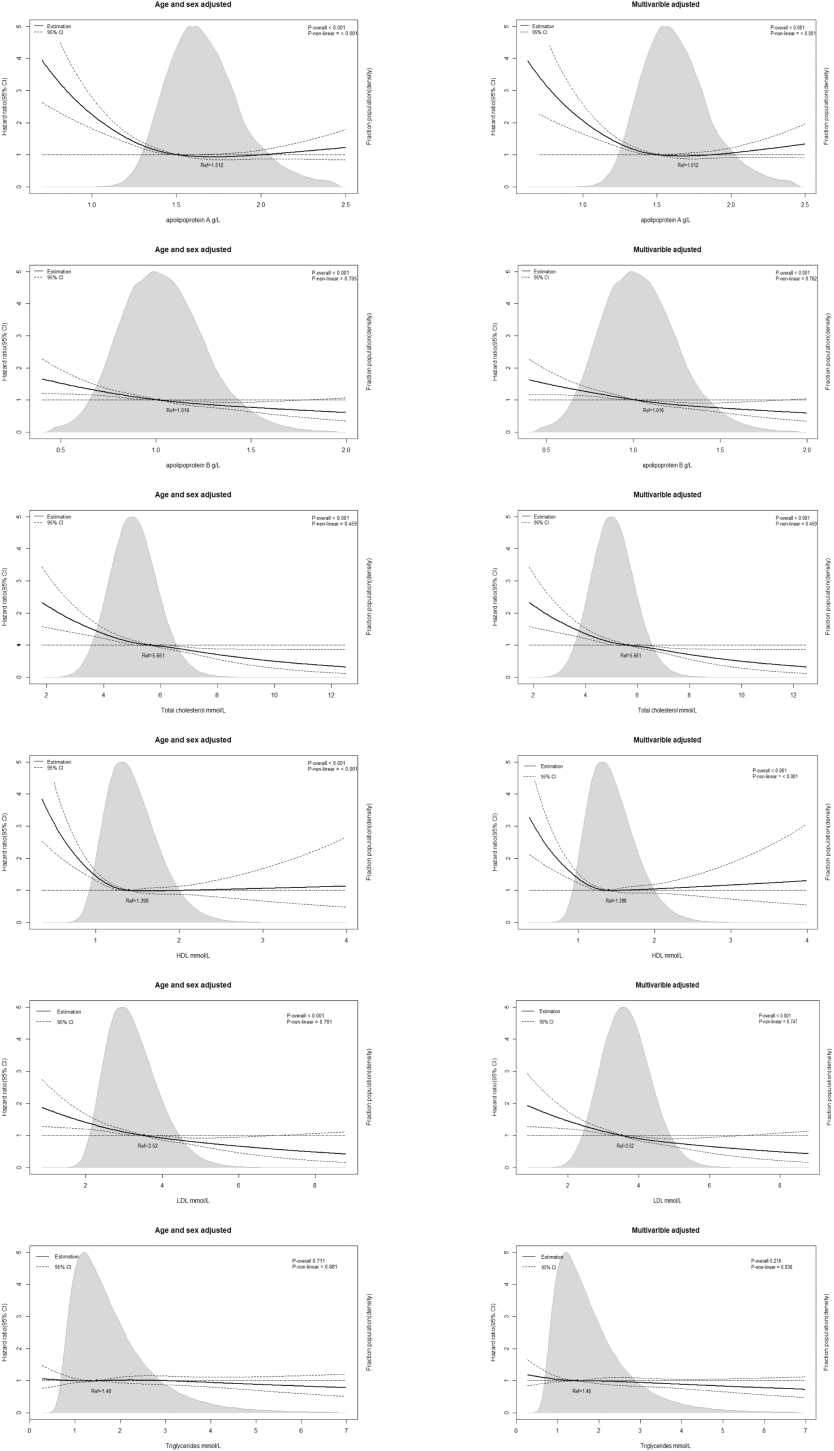
Association of ApoA, ApoB, total cholesterol, HDL, LDL levels and triglycerides level with risk of plasma cell neoplasms in individuals from the UK Biobank Study. Hazard ratios and 95% confidence intervals (CIs) were obtained from Cox proportional hazards regression with restricted cubic splines. Multivariable adjustment included age, sex, body mass index, ethnicity, alcohol taking frequency, qualification, diabetes status, smoking status, Townsend deprivation index and lipid‐lowering therapy. The median values of each lipid parameters were chosen as reference. The black line represents the hazard ratio and the dotted lines 95% CIs. Areas of gray represent the distribution of levels of each lipid parameters. *p*‐overall is calculated each parameter as a continuous variable in Cox model. *p*‐non‐linear was calculated by Wald Chi‐square test.

Figure 1 shows that all lipid profiles we analyzed in this study were inversely associated with plasma cell neoplasm risk (all *p*_trend_ <0.005). Total cholesterol, LDL, and ApoB level show a linear association with the risk of neoplasms (both *p*_nonlinear_ >0.05). However, HDL and ApoA level shows a nonlinear association (both *p*_nonlinear_ <0.05). Multivariable Cox proportional hazards regression was adjusted for sex, age, BMI, smoking status, alcohol taking frequency, qualification, Townsend deprivation index, metabolic Equivalent Task (MET) scores, plasma triglycerides, and lipid‐lowering medications with restricted cubic splines. In multiple models adjusted for different covariates, higher quartiles of baseline lipid levels were associated with a decreased risk of plasma cell neoplasms compared to the lowest quartile group. The adjusted HR was 0.82 (95%CI, 0.71–0.95), 0.67 (95%CI, 0.58–0.77), 0.71 (95%CI, 0.61–0.82), 0.66 (95%CI, 0.64–0.88), 0.79 (95%CI, 0.68–0.9) and 0.92 (95%CI, 0.79–1.07) for ApoA, ApoB, TC, LDL, HDL, and triglycerides, respectively, in model 3 (Table 2). However, the second low total cholesterol group and LDL group showed no significant decrease in plasma cell neoplasms risks. The ratio of lipid parameters were also analyzed(Supplementary Table S1). HDL/LDL ratio had no significance across all three models analysis. For HDL/ApoA ratio, the HRs in age‐ and sex‐adjusted model were 0.84 (95%CI, 0.72–0.96) when the highest quantile compared to the lowest quantile. For LDL/ApoB ratio, the third quantile (1.34–1.4) were associated with lowest risk of plasma neoplasm when compared with the lowest quartile and HR was 0.80 (95%CI, 0.69–0.92) in sex‐ and age‐adjusted model.

**TABLE 2:** Associations of serum lipids with plasma cell neoplasms in UK Biobank when excluding participants within 1‐year follow‐up.

	Lipid level		*N*	Case	HR					
					Model 1^a^	*p*	Model 2^b^	*p*	Model 3^c^	*p*
ApoA			406,903	1819						
	<1.347		101,393 (24.9%)	567 (31.2%)	Ref		Ref		Ref	
	1.347–1.512		101,793 (25.0%)	438 (24.1%)	0.78 (0.68–0.88)	<0.0001	0.80 (0.71–0.91)	0.0007	0.80 (0.71–0.91)	0.0007
	1.512–1.7		102,127 (25.1%)	398 (21.9%)	0.71 (0.63–0.82)	<0.0001	0.68 (0.59–0.8)	0.0001	0.76 (0.66–0.87)	<0.0001
	>1.7		101,590 (25.0%)	416 (22.9%)	0.76 (0.66–0.87)	<0.0001	0.82 (0.71–0.95)	0.008	0.82 (0.71–0.95)	0.0082
*p* for trend^d^					<0.0001		0.008		0.0082	
ApoB										
	<0.863		101,592 (25.0%)	567 (31.2%)	Ref		Ref		Ref	
	0.863–1.016		101,240 (24.9%)	464 (25.5%)	0.85 (0.75–0.96)	0.0079	0.86 (0.76–0.98)	0.0205	0.84 (0.74–0.96)	0.0087
	1.016–1.181		102,464 (25.2%)	400 (22.0%)	0.71 (0.62–0.8)	<0.0001	0.73 (0.64–0.83)	<0.0001	0.70 (0.61–0.8)	<0.0001
	>1.181		101,607 (25.0%)	388 (21.3%)	0.68 (0.6–0.77)	<0.0001	0.7 (0.61–0.79)	<0.0001	0.67 (0.58–0.77)	<0.0001
*p* for trend^d^					<0.0001		<0.0001		<0.0001	
Total cholesterol										
	<4.909		101,567 (25.0%)	578 (31.8%)	Ref		Ref		Ref	
	4.909–5.651		101,619 (25.0%)	452 (24.8%)	0.86 (0.76–0.98)	0.0204	0.90 (0.79–1.02)	0.0885	0.87 (0.77–0.99)	0.0409
	5.651–6.422		101,929 (25.1%)	402 (22.1%)	0.75 (0.66–0.85)	<0.0001	0.79 (0.69–0.9)	0.0004	0.76 (0.66–0.87)	0.0001
	>6.422		101,788 (25.0%)	387 (21.3%)	0.70 (0.61–0.8)	<0.0001	0.74 (0.64–0.85)	<0.0001	0.71 (0.61–0.82)	<0.0001
*p* for trend^d^					<0.0001		<0.0001		<0.0001	
LDL										
	<2.948		101,476 (24.9%)	576 (31.7%)	Ref		Ref		Ref	
	2.948–3.52		101,768 (25.0%)	463 (25.5%)	0.87 (0.77–0.99)	0.0295	0.90 (0.79–1.01)	0.0841	0.86 (0.76–0.98)	0.0251
	3.52–4.119		101,923 (25.0%)	402 (22.1%)	0.74 (0.65–0.84)	<0.0001	0.77 (0.67–0.87)	0.0001	0.72 (0.63–0.83)	<0.0001
	>4.119		101,736 (25.0%)	378 (20.8%)	0.68 (0.6–0.77)	<0.0001	0.70 (0.61–0.8)	<0.0001	0.66 (0.64–0.88)	<0.0001
*p* for trend^d^					<0.0001		<0.0001		<0.0001	
HDL										
	<1.171		101,558 (25.0%)	555 (30.5%)	Ref		Ref		Ref	
	1.171–1.398		101,551 (25.0%)	459 (25.2%)	0.87 (0.76–0.99)	0.0361	0.86 (0.76–0.99)	0.0298	0.86 (0.76–0.99)	0.0302
	1.398–1.674		102,339 (25.2%)	394 (21.7%)	0.84 (0.74–0.95)	0.008	0.82 (0.72–0.94)	0.004	0.82 (0.72–0.94)	0.004
	>1.674		101,455 (24.9%)	411 (22.6%)	0.82 (0.72–0.93)	0.0027	0.78 (0.68–0.9)	0.0006	0.79 (0.68–0.9)	0.0007
*p* for trend^d^					0.009		0.0025		0.0025	
Triglycerides										
	<1.043		101,689 (25.0%)	448 (24.6%)	Ref		Ref		Ref	
	1.043–1.48		101,716 (25.0%)	451 (24.8%)	0.87 (0.77–0.99)	0.03	0.91 (0.8–1.03)	0.1264	0.91 (0.8–1.03)	0.131
	1.48–2.142		101,781 (25.0%)	463 (25.5%)	0.77 (0.68–0.89)	0.0002	0.83 (0.72–0.95)	0.0073	0.83 (0.72–0.95)	0.008
	>2.142		101,717 (25.0%)	457 (25.1%)	0.84 (0.73–0.96)	0.0127	0.92 (0.79–1.07)	0.2828	0.92 (0.79–1.07)	0.2982
*p* for trend^d^					0.0085		0.2495		0.2658	

^a^ Adjusted model 1: Age and sex were adjusted.

^b^ Adjusted model 2: BMI, ethnicity, alcohol taking frequency, qualification, diabetes status and smoking status were additionally adjusted.

^c^ Adjusted model 3: cholesterol‐lowering medication is additionally adjusted.

^d^ The linear trend test was performed by using the median of each lipid category as an ordinal variable.

### 3.3 Subgroup analysis

**FIGURE 2:**
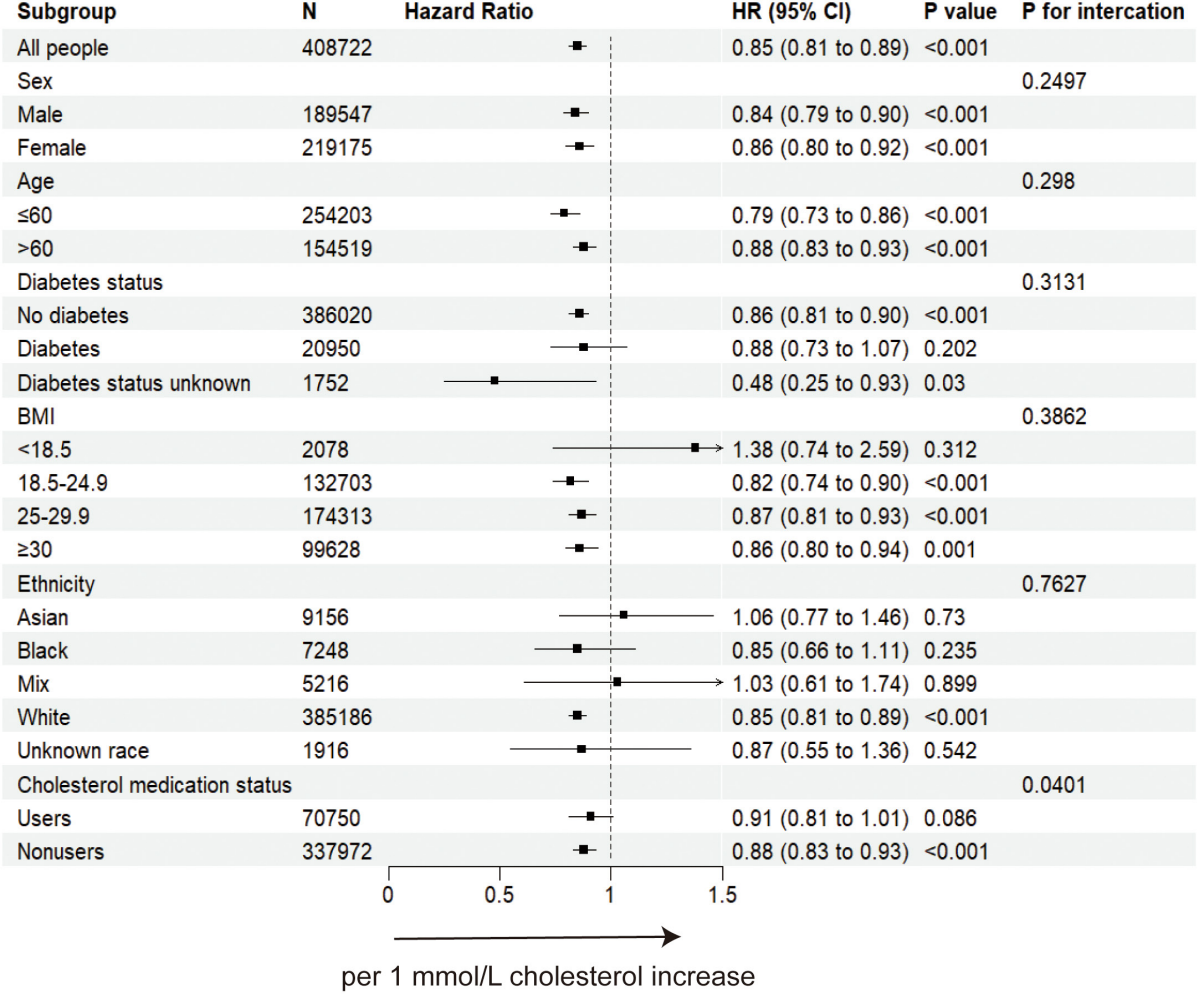
The forest plot showing the association between total cholesterol increase and plasma cell neoplasms incidence when excluding participants within 1‐year follow‐up. BMI, body mass index; CI, confidence interval; HR, hazard ratio. Cox proportional hazard model using age as the time scale and stratified by covariates including age, sex, body mass index, ethnicity, alcohol intake, qualification, diabetes status, smoking status, Townsend deprivation index and lipid‐lowering therapy.

**FIGURE 3:**
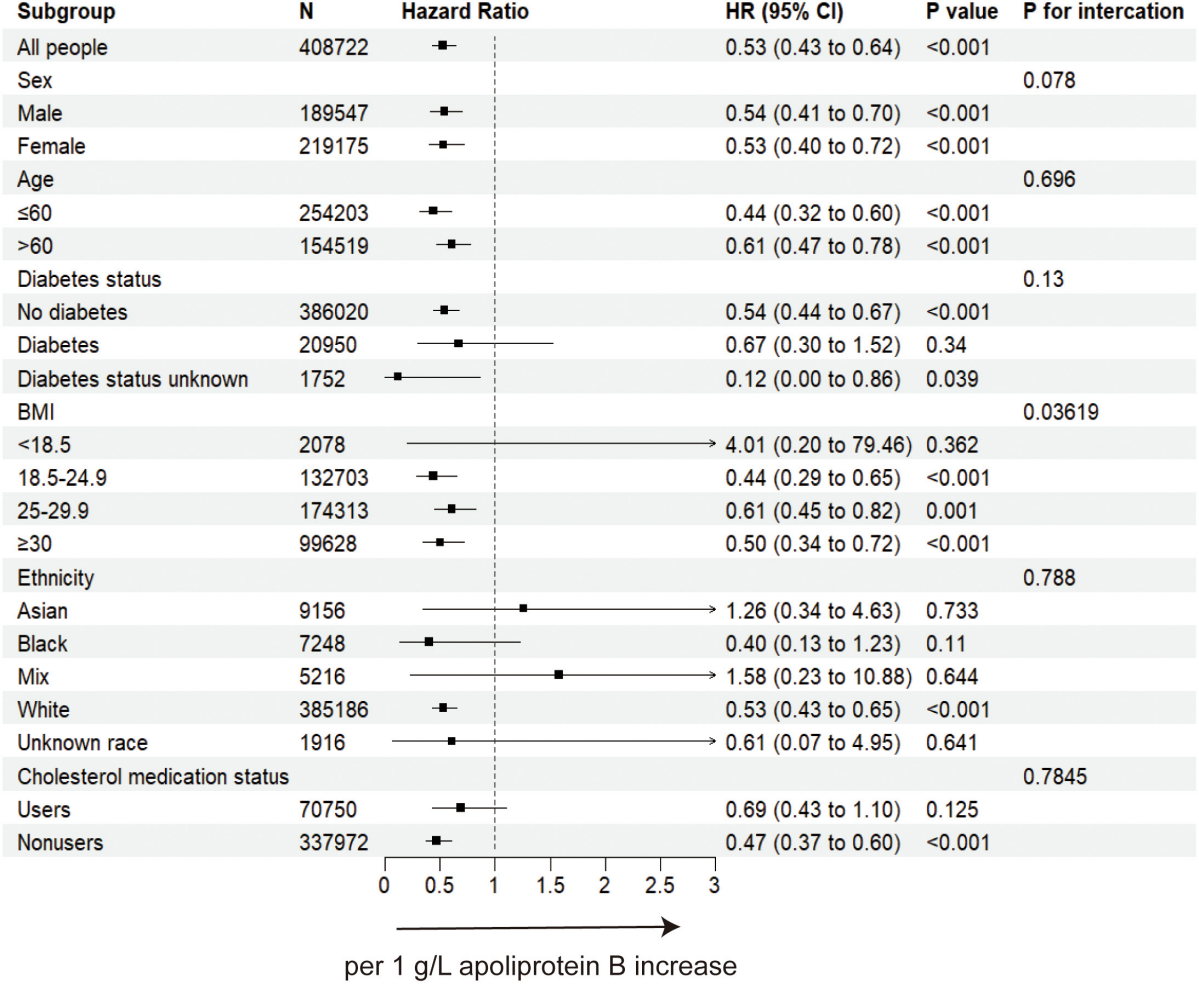
The forest plot showing the association between ApoB increase and plasma cell neoplasms incidence when excluding participants within 1‐year follow‐up. BMI, body mass index; CI, confidence interval; HR, hazard ratio. Cox proportional hazard model using age as the time scale and stratified by covariates including age, sex, body mass index, ethnicity, alcohol intake, qualification, diabetes status, smoking status, Townsend deprivation index and lipid‐lowering therapy.

The higher plasma cell neoplasms risk associated with lower cholesterol lipid level in the overall population was generally observed across sex, BMI, age, ethnicity, diabetes status, and lipid‐lowering medication.(Figures 2 and 3, Supplementary Figures S2–S4) Moreover, significant interactions across sex and age, and ApoA level were observed, with a higher risk in male and age over 60 years old populations. (*p*_interaction_ = 0.032 and 0.026, respectively). Significant modification effects by cholesterol medication were detected in total cholesterol analysis (*p*_interaction_ = 0.0401) and this suggest the inverse relationship between cholesterol level and plasma cell neoplasm may not observed in population taking cholesterol‐lowering medications as *p*‐value was 0.086 in users group.

### 3.4 Sensitive analysis

Results of sensitivity analysis by Cox proportional hazards model analysis were all consistent with principal findings in the population when excluding incident plasma cell neoplasms cases within three years after baseline (Supplementary Tables S2 and S3).

### 3.5 Mendelian randomization

MR‐Egger and inverse variance weighted mode did not show any statistical significance for ApoA, HDL, LDL, and total cholesterol levels for a causal association with the incidence of multiple myeloma. (Table 3) There was no suggestive association of genetically predicted serum ApoA, LDL, HDL, and total cholesterol levels with multiple myeloma.

**TABLE 3:** MR analyses of causal associations of lipid levels with multiple myeloma.

Exposure		MR‐IVW		MR‐Egger	
Lipid parameter	nSNP	*b*	*p*‐value	*b*	*p*‐value
LDL cholesterol					
met‐c‐895	19	2.31 × 10^−4^	0.253	3.96 × 10^−4^	0.222
bbj‐a‐31	21	4.50 × 10^−4^	0.094	3.25 × 10^−4^	0.361
ieu‐a‐300	73	2.10 × 10^−4^	0.337	2.25 × 10^−4^	0.490
Apolipoprotein A					
met‐c‐842	10	6.59 × 10^−5^	0.836	8.45 × 10^−5^	0.927
HDL cholesterol					
bbj‐a‐24	47	6.33 × 10^−5^	0.788	2.38 × 10^−4^	0.560
ieu‐a‐299	84	4.72 × 10^−4^	0.361	4.30 × 10^−4^	0.117
Total cholesterol					
met‐c‐933	17	3.23 × 10^−4^	0.230	4.53 × 10^−4^	0.371
bbj‐a‐54	37	3.53 × 10^−4^	0.325	2.23 × 10^−4^	0.707
ieu‐a‐301	82	2.47 × 10^−4^	0.271	3.64 × 10^−4^	0.130

Abbreviations: IVW, inverse variance weighted; MR, Mendelian randomization; nSNP, number of single‐nucleotide polymorphism.

## 4 DISCUSSION

We examined the associations between lipid metabolites and the risk of plasma cell neoplasms in this analysis from the UK Biobank. Overall, we found that lower levels of TC, HDL, LDL, ApoA, and ApoB were related to a higher risk of plasma cell neoplasms but triglyceride levels did not have the association. However, MR analysis results did not support the causal associations. The strengths of this study include (1) using both the restricted cubic spine model and quantile grouping model to identify the relationship between lipid level and plasma cell neoplasms risk, (2) using a sizable population‐based database of European ancestry, allowing analysis of relatively uncommon clinical outcomes like plasma cell neoplasms, (3) the first study to investigate potential causal associations of lipid level with plasma cell neoplasms using MR analyses even though the result was negative.

This study showed that there was an inverse relationship between HDL and ApoA level and the risk of plasma cell neoplasm, which agreed with what has been discovered in previous studies.^15^, ^17^ We also found that TC, LDL, and ApoB level, which were considered as bad particles unexpectedly presented the same effects as HDL and ApoA on the risk of plasma cell neoplasms. Although the mechanism underlying the low lipid level and risk of plasma cell neoplasms is not well understood, several potential explanations exist.

Several studies have revealed that HDL can regulate cancer cell proliferation.^26^, ^27^ Inflammatory pathways in cancer progression have also been found to be regulated by HDL through its anti‐inflammatory and antioxidative properties.^7^, ^28^ The presence of HDL and its ApoA could lead to a decrease in free cholesterol and lipid rafts in immune cells, consequently reducing inflammation and cell proliferation signaling pathways.^29^ The antitumorigenic effects of ApoA were clearly demonstrated in an in vivo study. The mechanism by which this occurs could have anticancer effects by indirectly altering the function of macrophages and other immune cells.^30^ Moreover, Scavenger receptor class B type (SR‐BI) plays an important molecule in the regulation of hematopoietic stem and progenitor cells' quiescence, differentiation, and proliferation through HDL‐related pathways.^31^ Furthermore, The expression of SR‐BI is significantly higher on tumor cell surfaces compared to other cell types. SR‐BI can promote the uptake of cholesteryl esters from HDL into the cytoplasm, resulting in a significant decrease in plasma HDL levels.^32^ Thus the low HDL level could also be the result of premalignant status. Our study shows that low HDL was associated with an increased risk of plasma cell neoplasms suggesting HDL could be essential in regulating the homeostasis of the hematopoietic system and its proliferation. The low level of HDL may increase the malignant transformation.

When it comes to LDL, an incongruous association between LDL and cancer risk has been found.^33^, ^34^ The relationship of LDL and total cholesterol level with cancer incidence differed markedly by cancer site.^35^ A few prospective epidemiological studies have revealed that there is an inverse association between serum LDL cholesterol level and the risk of cancer. However, these studies have been limited by some residual confounding factors and did not include hematological cancer.^33^, ^34^, ^35^

A low concentration of HDL cholesterol is associated with increased levels of oxidized LDL cholesterol. This, in turn, has been linked to higher levels of oxidative stress within cells,^36^ a process involved in cancer's pathogenesis.^37^ Thus, a low LDL level may produce less ox‐LDL with less oxidation harm to long‐lived plasma cells. However, recent in vitro study indicate that exogenous cholesterol is an essential metabolite for myeloma cells survival. On the other hand, low LDL plasma levels observed in patients with MM could be the consequence of the increased uptake of LDL by myeloma cells.^38^ Conversely, low HDL, LDL, and total cholesterol levels could be the consequences followed by cancer development. Low cholesterol levels, as suggested by some clinical studies and cases, may be also correlated with other hematologic malignancies such as chronic lymphocytic leukemia.^34^ Our result on MR indicates low lipid level did not have direct causal associations with MM, which means the low lipid level could be the secondary phenomenon or low lipid may regulate the biological process like apoptosis and oxidative stress affecting tumorigenesis.

More studies should be carried on to determine the clinical significance of this result, as the current knowledge of the specific relationship between lipid dysregulation and the development of plasma cell neoplasms is limited. Studies have shown that the take of statins in cancer patients has been linked to a reduction in cancer‐related mortality.^33^ A Swedish population‐based study found a 27% reduction in MM‐specific mortality associated with the take of statins within 6 months of diagnosis.^39^ In addition, the same result has been found in the American population‐based cohort study of patients with newly diagnosed MM. The take of statins was related to a 21% reduction in all‐cause mortality and a 24% reduction in MM‐specific mortality.^40^ However, according to our study compared to lipid‐lowering drug nonusers, the HR for users was 1.02(95%CI, 0.9–1.16), and *p*‐value was 0.7554 in multivariable‐adjusted Cox model. A recent mendelian randomization also found that lipid‐lowering therapies were not associated with multiple cancer risk including myeloma.^41^ Thus, taking cholesterol‐lowering drugs may not be helpful to decrease plasma cell neoplasm incidence, but the effect on multiple myeloma treatment and outcome need more clinical trial to find out. Therefore, in the future, further research on the risk of cancer in the treatment of dyslipidemia should be conducted. Furthermore, more research is required to determine the precise molecular mechanism connecting lipid levels with the carcinogenesis of plasma cell neoplasms.

We also noticed the albumin level differs between plasma cell neoplasm cases and noncases groups. We did the analysis of albumin levels, and compared to lowest quantile (<43.51 g/L) the HRs was 0.72 (95%CI, 0.63–0.82), 0.0.63 (95%CI, 0.54–0.72), 0.0.59 (95%CI, 0.51–0.69) for the second (43.51–45.21 g/L), third (45.21–46.94 g/L) and highest quantile (>46.94 g/L), respectively, in multivariable adjusted Cox model. The result is similar as the cholesterol levels. Some studies also have found that there was an inverse association between albumin level and cancer incidence.^42^ Furthermore, albumin level and cholesterol level together can represent ones' nutritional status. Malnutritional status could affect the immune system and lead to tumorigenesis.^43^

This study has some limitations. First, it only considers the lipid level of the baseline when the population is included in the study. The lipid variation is not considered in this study which may cause bias in the result. Second, some rare plasma cell neoplasm were not included in our study like POEMS syndrome owing to imprecise ICD‐10 code for such disease. Third, the prevalence of the plasma cell neoplasm varies from different countries and the study only included participants in UK. In addition, some risk factor like virus infection and autoimmune disease were not included in adjusted model and these factors may have influence on our results. Last, the incidence of the plasma cell neoplasm was relatively low compared to previous study.^44^ The reason for this low incidence in UK Biobank study may due to MGUS is an asymptomatic neoplasm and most elder people who have monoclonal gammopathy is difficult to be diagnosed as immunofixation electrophoresis was not an extensive screening test.

## AUTHOR CONTRIBUTIONS

**Linfeng Li:** Conceptualization (lead); formal analysis (equal); methodology (equal); writing – original draft (equal). **Zhengyu Yu:** Formal analysis (equal); funding acquisition (equal); software (equal). **Jianjun Ren:** Conceptualization (equal); project administration (lead); resources (lead); supervision (equal); writing – review and editing (equal). **Ting Niu:** Funding acquisition (equal); methodology (equal); project administration (equal); supervision (lead); writing – review and editing (equal).

## CONFLICT OF INTEREST STATEMENT

The authors declare that the research was conducted in the absence of any commercial or financial relationships that could be construed as a potential conflict of interest.

## ETHICAL APPROVAL STATEMENT

The data accession of the study was approved under the UK Biobank application (Applicant Number: 69718 and 80787). UK Biobank ethical approval was from the North West Multi‐centre Research Ethics Committee.

## Supporting information


Data S1.

## Data Availability

Data accession in this study can be applied from the UK Biobank by giving a request proposal.
