# Supplementary material for: Low cholesterol levels are associated with increasing risk of plasma cell neoplasm: A UK biobank cohort study
Source: Cancer Med. 2023 Nov 1;12(22):20964–75. doi: 10.1002/cam4.6649 (PMC10709719; doi:10.1002/cam4.6649)
Supplement: Supplementary file 1 — Data S1. [file CAM4-12-20964-s001.docx]

**Supplementary Figure 1.** Overview of study participants exuclucion and selection for this study. The selection criteria is shown in the box. A total of 408,722 eligible participants were eventually included in our study.

*N* = 502,507

Exclusion:

Withdrawal from the study (*n* = 76)

*N* = 502,494

Exclusion :

1.Prior diagnosis of any cancer except non-melanoma skin cancer at baseline (*n* = 25,802);

2.No serum lipid biomarker assessment and BMI at baseline (*n* = 63,215);

*N* = 413,477

Exclusion:

Participants with ≤ 1years of follow-up (*n* = 4,775)

*N* = 408722

Plasma cell malignancy cases (*n* = 1819)

Non-cases (*n* = 406903)

**Supplementary Figure 2.** The forest plot showing the association between ApoA increase and plasma cell neoplasms incidence when excluding participants within 1-year follow-up.


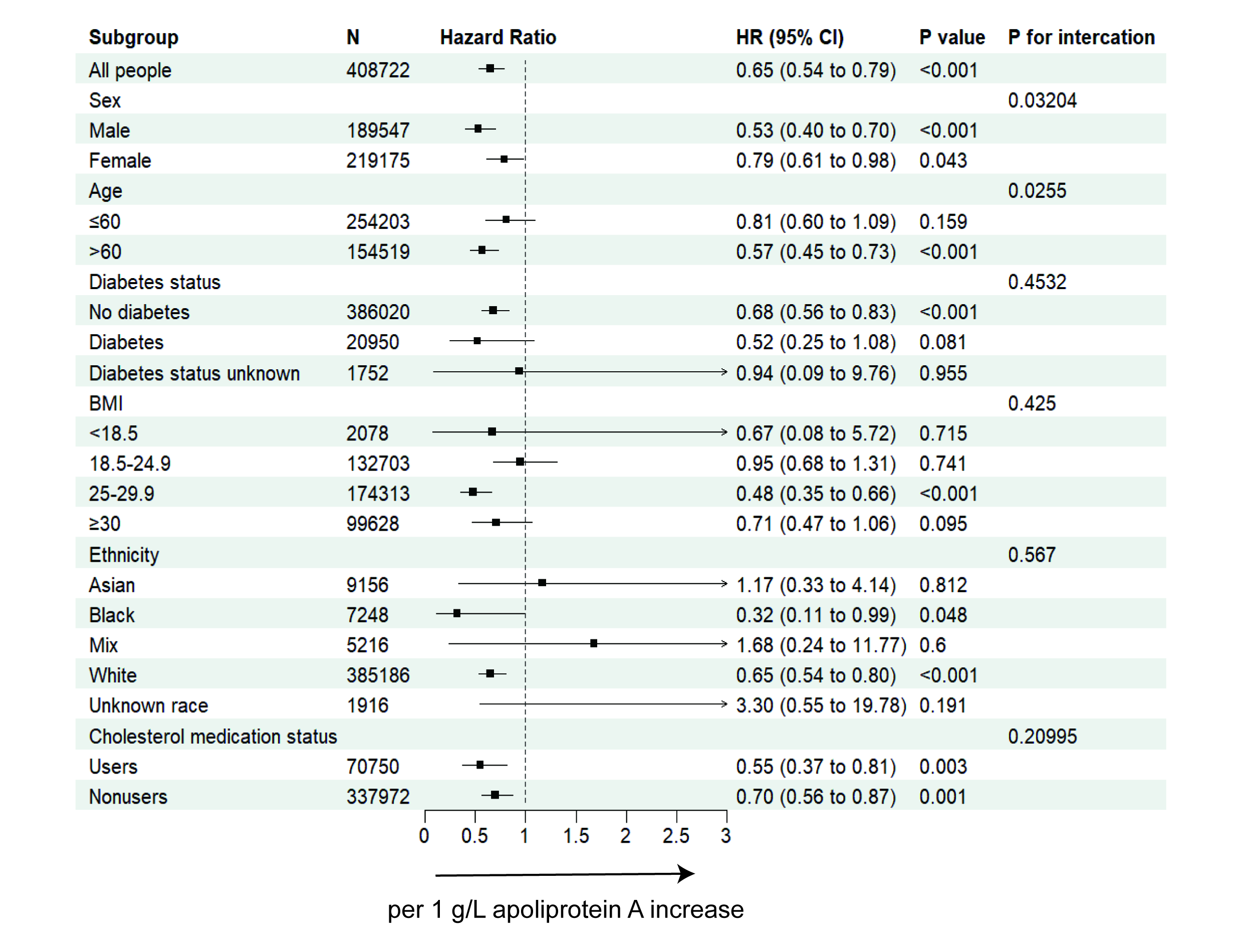


Abbreviations: CI, confidence interval; ApoA, apoliprotein A; HR, hazard ratio;BMI, body mass index.

Cox proportional hazard model using age as the time scale and stratified by covariates including age, sex, body mass index, ethnicity, alcohol taking frequency, qualification, diabetes status, smoking status , townsend deprivation index and lipid-lowering therapy.

**Supplementary Figure 3.** The forest plot showing the association between HDL increase and plasma cell neoplasms incidence when excluding participants within 1-year follow-up


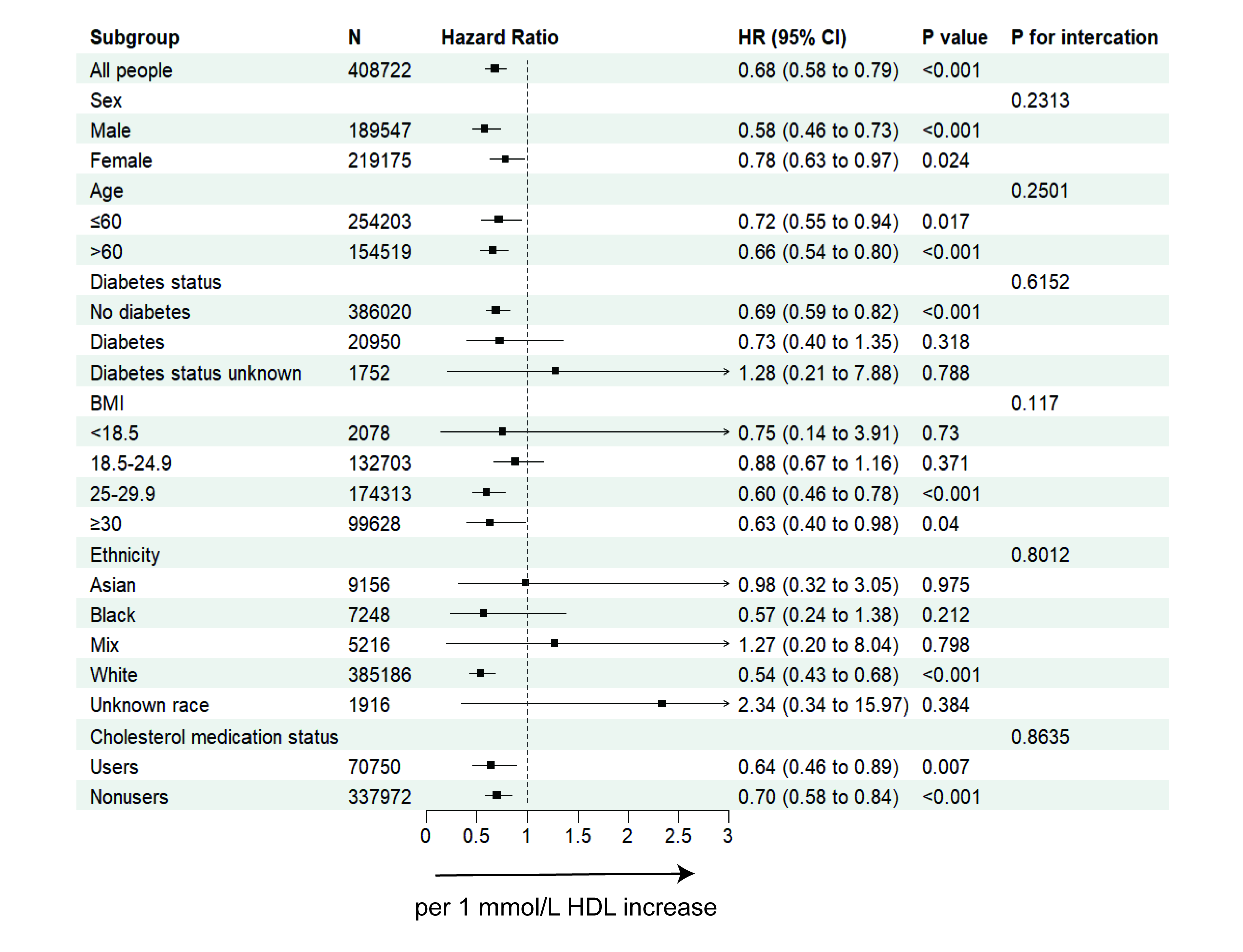


Abbreviations: CI, confidence interval; HDL, high-density lipoprotein; HR, hazard ratio;BMI, body mass index.

Cox proportional hazard model using age as the time scale and stratified by covariates including age, sex, body mass index, ethnicity, alcohol taking frequency, qualification, diabetes status, smoking status , townsend deprivation index and lipid-lowering therapy.

**Supplementary Figure 4.** The forest plot showing the association between LDL increase and plasma cell neoplasms incidence when excluding participants within 1-year follow-up


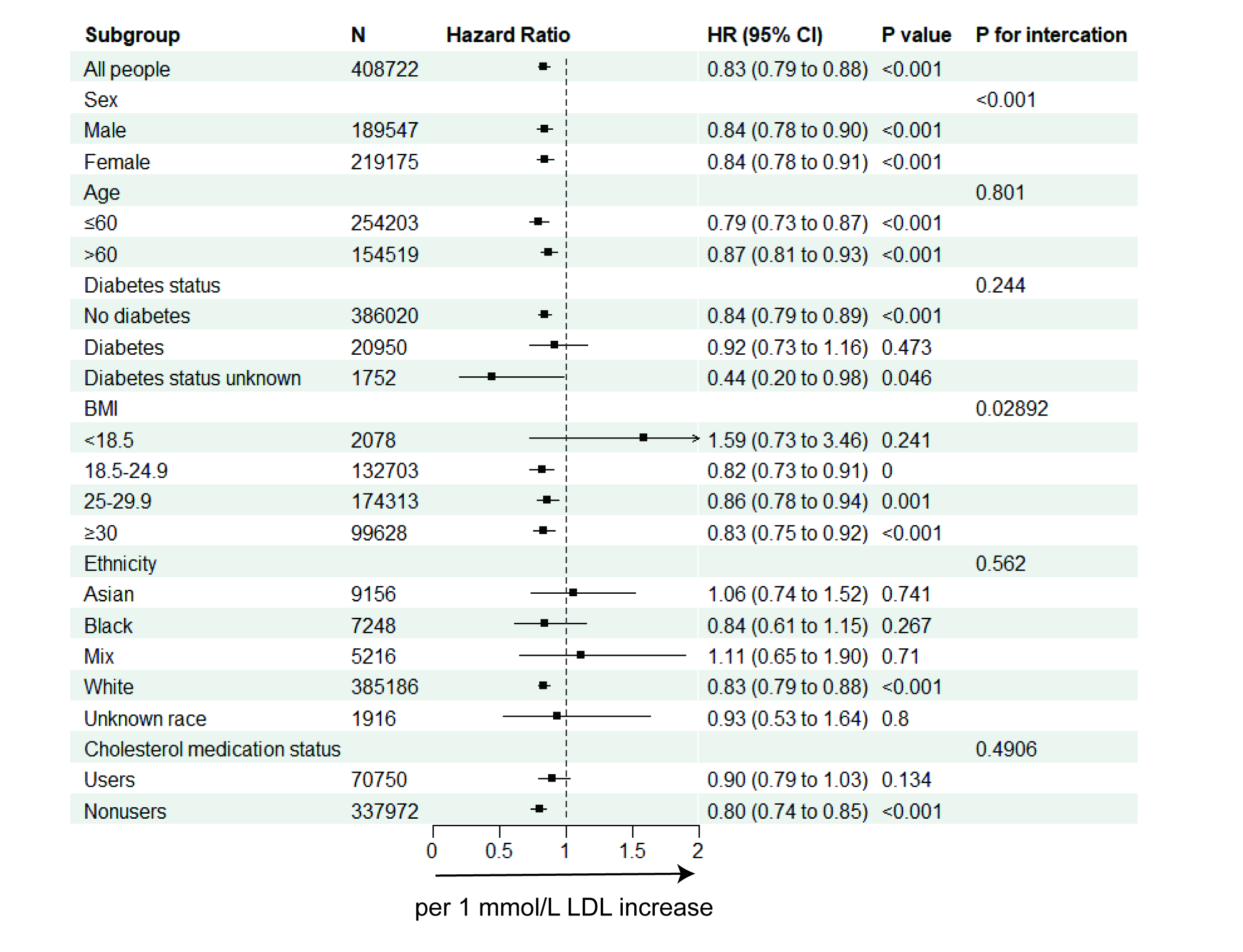


Abbreviations: CI, confidence interval; LDL, low-density lipoprotein; HR, hazard ratio;BMI, body mass index.

Cox proportional hazard model using age as the time scale and stratified by covariates including age, sex, body mass index, ethnicity, alcohol taking frequency, qualification, diabetes status, smoking status , townsend deprivation index and lipid-lowering therapy.

**Supplementary Table 1. Associations of serum lipids ratios with plasma cell neoplasms in UK Biobank when excluding participants within 1-year follow-up**

The linear trend test was performed by using the median of each lipid category as an ordinal variable.

Adjusted model 1: Age and sex were adjusted; Adjusted model 2: BMI, ethnicity, alcohol taking frequency, qualification, diabetes status and smoking status were additionally adjusted. Adjusted model 3: cholesterol lowering medication is additionally adjusted.

**Supplementary Table 2.** Baseline characteristics of individuals in UKB excluding participants within 3-year follow-up

a: P values were determined from Wilcoxon rank-sum test to compare differences for continuous variables and Pearson test for categorical variables between cases

and non-cases.

b: Median alcohol taking frequency is defined as frequency less than twice a week and heavy alcohol taking frequency is defined as frequency over three times a week.

**Supplementary Table 3. Associations of serum lipids with plasma cell neoplasms in UK Biobank when excluding participants within 3-year follow-up**

The linear trend test was performed by using the median of each lipid category as an ordinal variable.

Adjusted model 1: Age and sex were adjusted; Adjusted model 2: BMI, ethnicity, alcohol taking frequency, qualification, diabetes status and smoking status were additionally adjusted. Adjusted model 3: cholesterol lowering medication is additionally adjusted.
